# A visual perceptual perspective on gaze in social robotics

**DOI:** 10.3758/s13423-026-02902-x

**Published:** 2026-04-02

**Authors:** Roy S. Hessels, Yu Fang

**Affiliations:** 1 Experimental Psychology, Helmholtz Institute, Utrecht University, Heidelberglaan 1, 3584CS Utrecht, The Netherlands; 2 Honda Research Institute Japan Co., Ltd., 8-1 Honcho, Wako-shi, Saitama 351-0188 Japan

**Keywords:** Human interaction, Social robotics, Gaze, Eye tracking, Visual perception

## Abstract

Where and when should a robot look during interaction with humans? Intuitively, human–human interaction serves as the blueprint. In such interactions, gaze plays a central role, both in visually guiding action and in signaling communication. Yet, most social robotics research still relies on the oversimplified eye–mind assumption, equating gaze with visual attention, and often implements gaze behaviors without accounting for perceptual limits or individual variability. We identify three core challenges at the intersection of psychology, visual perception, and robotics: (1) disentangling the dual functions of gaze, (2) understanding the visual perceptual mechanisms underlying gaze perception, and (3) adapting to individual, cultural, and clinical variability in gaze behavior. By examining these challenges in both robot interpretation and production of gaze, we argue that social robotics benefits from insights into human gaze and provides a powerful testbed for theory development, enabling controlled, interactive experiments that deepen our understanding of social attention. We conclude by conceptualizing social gaze as a soft-constraints inference and production process under uncertainty, linking visual perceptual mechanisms and social meaning.

How does one design a social robot that interacts naturally with humans? Intuitively, one takes human–human interaction as a blueprint for the development of human–robot interaction. Human interaction is inherently multimodal, involving multiple sensory and communicative modalities (auditory, visual, haptic, etc.). Among the modalities, gaze plays a key role in human interaction, both as a perceptual system guiding action and as a channel for nonverbal communication. Yet, most socially interactive systems still rely on the “eye–mind” assumption, equating gaze with visual attention, an approach contested both in general (Hessels et al., 2025b) and specifically in the case of social gaze by research on, e.g., covert attention (Foulsham & Kingstone, 2025) and cross-cultural variation in gaze norms. Here, we argue that social robotics provides a unique empirical and theoretical testbed for studying the dual functions of gaze, while perceptual and psychological theories provide the foundations for designing meaningful, interpretable behaviors. Unlike humans, who have a lifetime of learning when and where to look, robots must explicitly interpret and produce gaze behaviors. This engineering necessity forces us to explicitly address unresolved questions in psychology and perception research, especially on when gaze serves overlapping or conflicting functions. Social robotics, therefore, is not just an application area, but may reveal a new understanding of social gaze through human–robot interaction paradigms.

From the intersection of visual perception, eye-movement research, and experimental psychology, we identify three core challenges for future gaze modeling and interpretation in social robotics, with theoretical relevance to psychology: (1) the dual function of gaze; (2) the perceptual mechanisms underlying gaze perception, from foveal versus peripheral vision to fine-grained direction judgments; and (3) moving from broad empirical laws to systems that adapt to individual, clinical, and cultural differences. For each, we address both robot interpretation and robot production of gaze, and conclude by closing the loop from human–human interaction, through human–robot interaction, back to theory development. Throughout this paper, we focus on humanoid and anthropomorphic robots designed for face-to-face social interaction, where gaze behavior is realized through human-like eyes, head orientation, or face-like geometry. Our arguments therefore do not necessarily generalize to non-humanoid platforms, for which gaze cues may be absent or functionally different.

The necessary foundation for our perspective comes from classical theories of gaze processing in humans as well as important, yet often overlooked empirical research on the perception of gaze direction. Theoretically, for example, early models by Baron-Cohen (1997) proposed specialized mechanisms such as the Eye-Direction Detector (EDD) and Shared Attention Mechanism (SAM), through which humans compute other people’s spatial attention and infer mental states. Empirically, the perceptual and communicative dimensions of gaze, e.g., how gaze direction is perceived, how gaze can trigger shifts of attention and how intentionality transforms gaze into a social signal, are further well established (see e.g., Gibson & Pick, 1963; Driver et al., 1999; Emery, 2000; Langton et al., 2004; Frith & Frith, 2006; Frischen et al., 2007; Kingstone, 2009). While these frameworks have laid the groundwork for understanding gaze as both a low-level perceptual cue and a high-level communicative act, we focus on how these concepts must now be explicitly operationalized for social robotics. Specifically, we have two aims: (1) to integrate a diverse set of classical theoretical and empirical insights from psychology and visual perception research into a coherent set of challenges for the social robotics field, and (2) to serve as a bridge between psychological research and social robotics, linking empirical research in both fields and promoting closer interdisciplinary collaborations. The latter goal is especially important given that scientific (publication) cultures may vary tremendously between disciplines, which in our experience may mean that important research in either discipline is overlooked by the other.

Note that in human interaction, gaze production and interpretation are tightly coupled with linguistic information, including speech content and turn-taking cues (e.g. Kendon, 1967; Clark, 1996). Verbal context can constrain how gaze directed toward or away from an interlocutor is interpreted. In the present paper, however, we deliberately focus on gaze as a visual perceptual signal, abstracting away from explicit linguistic modeling. This restriction allows us to isolate perceptual mechanisms and constraints that operate even in the absence of spoken language, and that remain essential for understanding gaze production and interpretation in both human–human and human–robot interaction.

## The dual function of gaze

That gaze serves both as a perceptual system guiding action, and as a channel for nonverbal communication, is known as the dual function of gaze (Gobel et al., 2015; Nasiopoulos et al., 2015; Risko et al., 2016). On the one hand, gaze serves an information-gathering function (Hayhoe & Ballard, 2005, 2014; Hayhoe, 2017). During sequential tasks (Cooper & Shallice, 2000; Botvinick & Plaut, 2004), humans move their eyes to acquire task-relevant information, closely linked in time to ongoing actions (Land et al., 1999; Hayhoe, 2000; Pelz & Canosa, 2001; Sullivan et al., 2021). On the other hand, gaze may serve an information-signaling function, regulating turn-taking, expressing intimacy, exercising social control, or aiding disambiguation (Argyle & Cook, 1976; Kendon, 1967; Argyle & Dean, 1965; Kleinke, 1986; Ho et al., 2015; Hessels et al., 2019; Maran et al., 2021; Hanna & Brennan, 2007; Macdonald & Tatler, 2013). While the dual function of gaze has long been acknowledged, we are unaware of theories or models that specify when these functions overlap, diverge, or conflict in practice. This gap is critical for social robotics, where systems must both interpret gaze, inferring what a human is looking at and their intended action or message, and produce gaze that conveys the intended information back to the human (Ruhland et al., 2015; Admoni & Scassellati, 2017).

### Interpretation

To engage in interaction with humans, social robots must interpret a range of (non-)verbal communicative cues. Unlike humans, robots require explicit models of gaze behavior to interpret the human’s intent. The goal of such models is to infer, e.g., what object a person is about to act upon, what action they will carry out, or whether they will start or stop talking (see, e.g., Zhang et al., 2020; Robinson et al., 2023; Belardinelli, 2024, for reviews). A human’s gaze location may be informative for intention prediction, simply because humans need to look at an object to manipulate it (gaze for perception) (e.g., Trick et al., 2019). Yet, gaze may also be used explicitly as a communicative signal, such as looking at one of multiple similar objects to disambiguate which is being addressed (e.g., Macdonald & Tatler, 2013). While both functions are well recognized, we are unaware of existing models in social robotics that explicitly distinguish between these two roles of gaze, perceptual and communicative, either as separate processes or as co-occurring signals. Most current approaches implicitly conflate these functions or treat them heuristically without systematic differentiation. For example, although recent works in visual attention modeling for robots (e.g., Ruesch et al., 2008; Hanifi et al., 2023; Pan et al., 2020) implement gaze-based perception or expression, they do not explicitly model the functional distinction between gaze-for-perception and gaze-for-communication.

Is explicit modeling of the dual functions of gaze essential? We believe it is. We propose that communicative gaze may not be an entirely separate function, but at least partially a byproduct of visual information processing: the very act of perceiving the world leaves observable traces in gaze behavior, becoming a powerful cue for others to infer intentions, emotions, and goals. In this view, communication through gaze emerges naturally from the public nature of perception, rather than being limited to deliberate expressive acts. A visual perceptual account would therefore address part of the communicative challenge by explaining when gaze unintentionally signals information. However, when gaze is used deliberately to communicate, robots must be able to detect this intent and respond appropriately (Lavit Nicora et al., 2024; Belardinelli, 2024; García-Martínez et al., 2025).

Additionally, social robots must infer human goals or interests even when overt gaze is suppressed. For example, in brief passing encounters, humans may suppress overt gaze to another person to avoid potential interactions (Hessels et al., 2020), or avoid following the direction of another person’s gaze when that person can observe them directly (Gallup et al., 2012). Similarly, in deceptive scenarios, humans may wish to intentionally hide their focus of attention to not give something away (Millen & Hancock, 2019). To navigate such scenarios, robots must go beyond interpreting gaze direction alone and incorporate multimodal communicative behaviors, context, and even social norms. These constraints push us to conceive more sophisticated models of human gaze behavior, ones that may ultimately shed light on covert attention in human–human interactions, where direct measurement remains elusive (Foulsham & Kingstone, 2025).

**Fig. 1:**
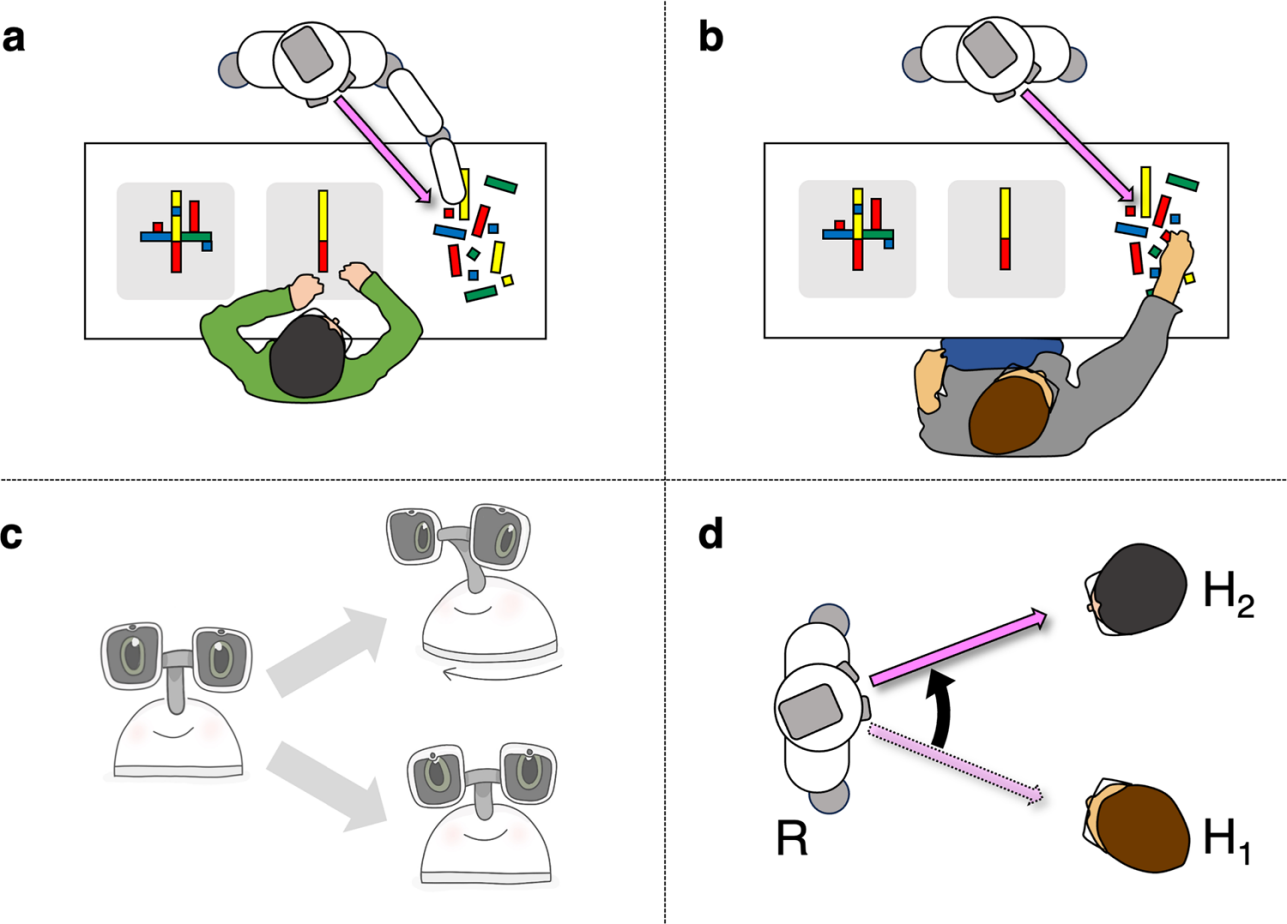
Example robot gaze behaviors. (**a**) Perceptually realistic gaze behavior: Robot demonstrating gaze-action coupling, gazing at the end point of the limb, for example, during a pointing gesture or object manipulation (based on Hessels et al., 2025a). (**b**) Perceptually realistic gaze behavior: Robot demonstrating action observation, gazing at an object being manipulated by the interacting human (based on Hessels et al., 2025a). (**c**) Bio-mechanically realistic gaze behavior: Robot demonstrating vestibulo-ocular reflexive eye movement during head movement (Fang et al., 2024). (**d**) Communicative gaze behavior: Robot (R) making a gaze shift from one human (H$_1$) to another (H$_2$) to regulate turn-taking (e.g., Mutlu et al., 2009)

### Production

A common approach in social robotics is to have robots replicate visible human behaviors in predesigned scenarios, such as two- or three-party conversation (Huang & Mutlu, 2012; Shintani et al., 2024). Yet, social robots allow for dissociation of gaze functions in a way that is not possible with humans. For example, a robot’s sensors are not necessarily coupled to its gaze, allowing it to look one way while its sensing systems ‘attend’ elsewhere. This opens several exciting experimental possibilities. First, researchers can ask whether producing perceptually realistic gaze leads to interactions that are perceived as more natural, without the need to concurrently consider communicative behavior. Producing perceptually realistic gaze may be achieved along several lines. At the behavioral level, it may include reproducing, e.g., visuomotor coordination and action observation (e.g., Land et al., 1999; Flanagan & Johansson, 2003; Hessels et al., 2025a), as illustrated in Fig. 1, panels A-B. However, it may also involve generating biomechanically realistic eye-movement behaviors. Pan et al. (2020), for example, found that an animatronic robot that produced saccadic eye movements increased perceived realism. They hypothesized that the produced saccades created an illusion of vergence and consequently a robot that looks ‘at you’, not ‘through you’. Similarly, Haefflinger et al. (2023) found that decoupled eye and head movements lead to a subjectively perceived higher ‘naturalness’ of the robot. In other work, Fang et al. (2024) demonstrated that implementing vestibulo-ocular-like coordination in a robot’s eye-head movement significantly increased users’ perceptions of direct gaze by the robot (illustrated in Fig. 1, panel C). Krüger et al. (2026) showed that enhancing screen-based 2D eye models with simulated corneal reflections, mimicking the subtle visual effects of real eyes, improved users’ ability to localize the robot’s referential gaze. These studies show that both biomechanical realism and visual augmentation can shape the effectiveness of robotic gaze in conveying attention and intent.

One may expect, however, that reproducing biomechanical realism alone does not ensure social meaning. Psychological and neurocognitive research suggests that gaze becomes communicative only when it is interpreted as intentional, that is, as evidence of an agent’s mental state or goal (e.g., Teufel et al., 2009; Wiese et al., 2012). As Kingstone (2009) argues, people care about the eyes of others and where those eyes are looking, but the cognitive processes associated with this depend tremendously on the situational context, for example, on the agency attributed to the gazer. This underscores that robotic gaze must not only appear natural in movement but also be perceived as arising from intentional control (see also Wykowska et al., 2016). In practical terms, this means designing systems that make their gaze goals interpretable and responsive to the environment, rather than merely realistic. In that regard, visuomotor coordination and action observation are excellent first candidates to convey intentionality through perceptually relevant gaze behavior.

An important long-term consideration is that gaze cues are not perceived in isolation from the robot’s overall appearance. The overall aesthetic design of the robot, including the degree of anthropomorphism, face shape, and facial feature design, may modulate affective responses such as trust or unease, which in turn may shape whether and how humans engage in eye contact. For example, too realistic facial designs may elicit discomfort or ‘uncanny valley’ effects, whereas pet- or toy-like designs may promote affiliation. Prior research suggests that while facial expressiveness and gaze can enhance perceived naturalness, the uncanny valley effect is more likely to arise from mismatches between gaze behavior, expressed affect, and attributed mental states than from gaze alone (Zheleva et al., 2023; Cihodaru-tefanache & Podina, 2025). Thus, from a visual-perceptual perspective, these aesthetic features act as contextual factors that may influence how gaze behavior is interpreted and responded to.

A second exciting experimental possibility is the explicit modeling of communicative functions above and beyond perceptual functions of gaze, and to investigate whether exaggerated or selectively omitted communicative gaze cues alter the human interpretation of a robot’s intent (e.g., extending the approach by Xu et al., 2016). Although isolated gaze cues in human-robot interaction are well studied, e.g., for turn-taking (Mutlu et al., 2009, see also Fig. 1, panel D), directing attention (Mwangi et al., 2018), object handovers (Moon et al., 2014), or reaching (Boucher et al., 2012), we argue for modeling perceptually realistic gaze first and then selectively adding or manipulating communicative functions. Finally, researchers can simulate covert attention in robots, creating conditions where gaze behavior intentionally or partially obscures internal states, and investigating how the robot is perceived. Social robotics thus provides a testbed for systematically evaluating how gaze production strategies, both perceptual and communicative, interact with human expectations and interpretations.

## Perception of gaze direction

An essential perceptual assumption regarding the interpretation and production of gaze by social robots is that humans are capable of accurately perceiving where others are looking, and thus a robot should do so too. Yet it may be that robotic systems do not perceive gaze direction with the same accuracy, or, conversely, that they do not suffer from the limitations in gaze perception that humans do. That begs the question of what the human perceptual limits are for perceiving another person’s gaze direction. To answer that question, it is important to realize the biological nature of human vision. While central vision, supported by the (para)fovea, provides high visual acuity at and around the point of fixation (Bringmann et al., 2018), lower-acuity vision is possible from the periphery (see, e.g., Rosenholtz, 2016; Loschky et al., 2019; Vater et al., 2022). The question can thus be reformulated into two questions (as outlined in Hessels, 2020): (1) what is the perceptual limit at foveal vision, and (2) what can be perceived without looking at something directly, i.e., from peripheral vision?

Regarding the perceptual limit at foveal vision (i.e., when one fixates the eyes of another person) of perceiving another person’s gaze direction, it has been estimated that humans are sensitive to iris displacements of less than 1 mm at 1–2-m inter-person distance (e.g., Gibson & Pick, 1963; Cline, 1967; Symons et al., 2004). More specifically, the perception of being looked at has been conceived as a cone of gaze of a looker, approximately 4-9$^{\circ }$ in width and distance-invariant (Gamer & Hecht, 2007; Horstmann & Linke, 2021). Thus, it seems that humans are exceptionally good at detecting when they are being looked at. However, it has also been shown that estimates of gaze direction are not perfect. For example, horizontal gaze direction tends to be perceived more to the side than it actually is (Anstis et al., 1969; Horstmann & Linke, 2025), and it can be biased by head direction (Langton et al., 2004; Kluttz et al., 2009). Moreover, it has been suggested that humans tend to assume they are being looked at, especially under uncertainty (e.g., Mareschal et al., 2013a, b).

For more fine-grained perception of gaze location within the face, relatively little empirical research seems to exist. von Cranach and Ellgring (1973) report in their review that deciding whether one is looked at in the eyes or the mouth could be assessed above chance, but not with very high accuracy ($$< $$50% correct at 80 cm, $$< $$30% at 200-cm inter-person distance for distinction of left vs. right eye, only 10% accuracy reported for 200-cm inter-person distance in another study). Moreover, the perception of being looked at in the eyes was not more accurate than for other areas within the face, and responses are likely biased towards the eyes. Relatedly, Chen (2002) reported that participants were less sensitive for deviations downward from the eyes when asked to assess whether someone was making eye contact with them. Thus, perceiving whether one is looked at in the mouth or eyes is more difficult than perceiving whether one is looked at and presumably depends on the inter-person distance. However, both being looked in the eye or in the mouth area can be interpreted as eye contact (see, e.g., Honma et al., 2012). Note that in research on gaze perception, terms such as direct gaze, mutual gaze, and eye contact are sometimes used interchangeably, sometimes as an operationalization for the other (e.g., mutual gazing at each other’s face as an operationalization for eye contact), and sometimes with objective or subjective meanings and operationalizations (see e.g. Heron, 1970; Honma et al., 2012; Jongerius et al., 2020).

Regarding what can be perceived from peripheral vision, several relevant studies have been conducted. Loomis et al. (2008) showed that accurate perception of head orientation changes can occur even in peripheral vision (up to 90$^{\circ }$ eccentricity). However, judging gaze direction from the eyes alone is more difficult: accuracy drops beyond 8$^{\circ }$ eccentricity at close viewing distances (84 cm) and beyond 4$^{\circ }$ at farther distances (300 cm). Similarly, Florey et al. (2015) found that, in peripheral vision, judgments of a person’s gaze direction rely primarily on head orientation rather than the eyes themselves. Thus, when not looking at another person directly, it is likely that judgements of gaze direction are determined by head direction, body orientation, or even the context, rather than the physical orientation of that person’s eyes in the world. Again, understanding these limitations is essential for social robots, who need to interpret attention, intentions, or communicative signals from eye, head, and body cues, and produce gaze that effectively conveys such information back to the human.

The here reviewed empirical studies of gaze perception also emphasize why studying embodied interactions with social robots is necessary instead of with, for example, virtual agents. The perceptual capabilities of peripheral vision exceed what is available from most screen-based studies. In addition, perceiving gaze direction for flat images can differ tremendously from that of physical agents (Horstmann & Linke, 2025), as exemplified by phenomena like the Mona Lisa effect (i.e., a picture of a face with forward-facing eyes appears to always look at the observer directly, regardless of where the observer stands). At the same time, many existing social robots employ 2D screens to present faces or eyes for reasons of cost, robustness, or expressive flexibility. The potential influence of the Mona Lisa effect thus has to be explicitly considered in such designs. For example, recent work has explored how augmenting screen-based eye models with additional visual cues, such as dynamic, reflection-like overlays contingent on eye movements, can reduce ambiguities associated with pupil-based pointing by providing scene-dependent spatial references (Krüger et al., 2026). Other work has demonstrated that coordinating a robot’s eye and head movements based on principles of the vestibulo-ocular reflex can enhance the perception of direct gaze during face-to-face interaction, even when the eyes are presented on 2D screens (Fang et al., 2024).

**Fig. 2:**
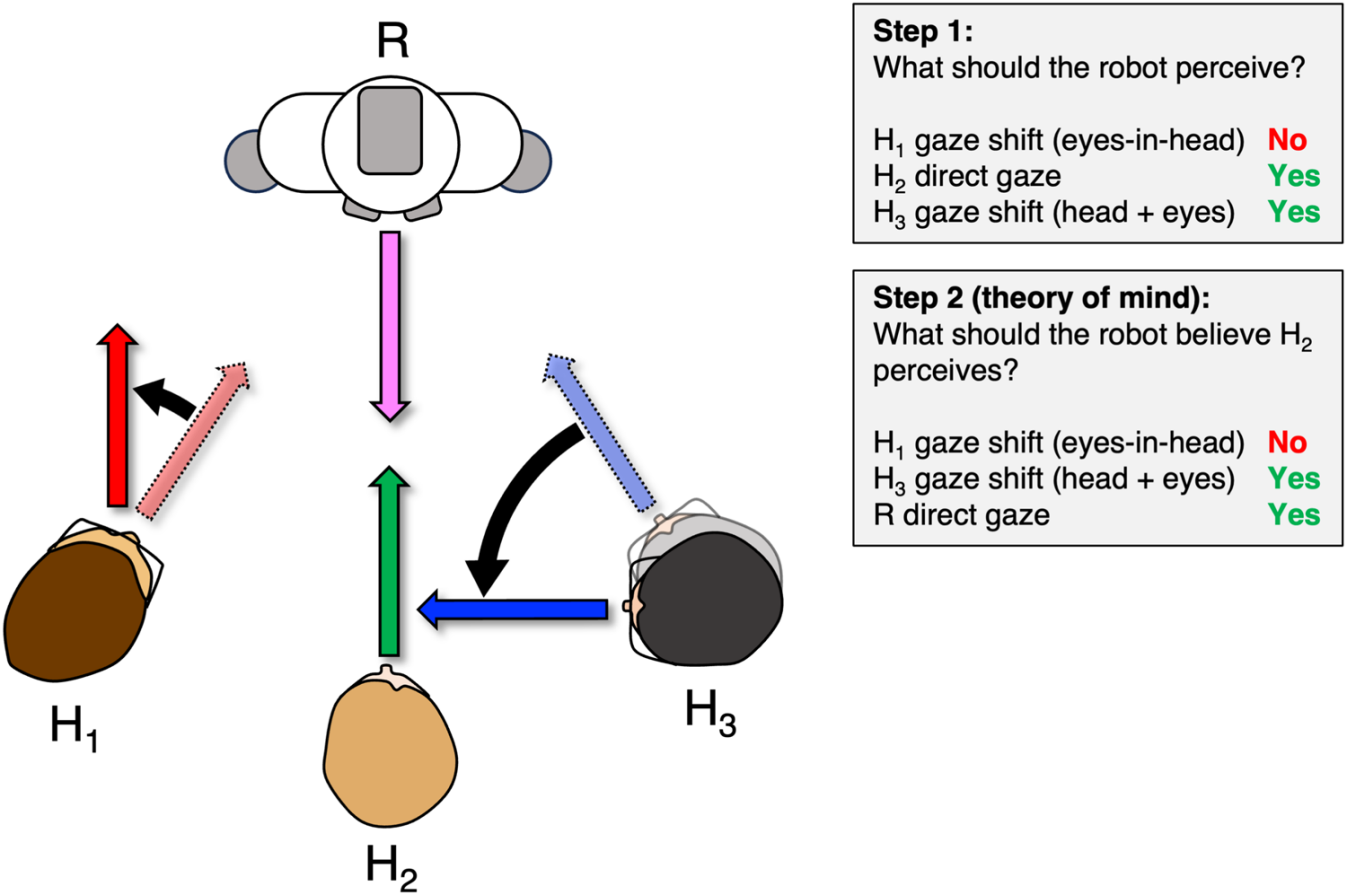
Human-informed gaze perception for social robots. The robot (R) and human 2 (H$_2$) maintain direct gaze. Human 1 (H$_1$) makes a gaze shift from the robot to a location in the background with their eyes only. Human 3 (H$_3$) makes a combined eye-head movement from the robot to H$_1$

### Interpretation

Given the exceptional human performance for estimating another person’s gaze direction in the world, one might expect social robots to be capable of the same. However, for social robots, interpreting where a human is looking in the environment is likely more important than estimating the exact direction of gaze when the human is looking at the robot. Like humans, robots may be expected to be adept at perceiving the human’s attentional target (e.g., at what object they may interact with), as this can signal intention or upcoming behavior. In contrast, distinguishing whether the human is looking at the robot’s eyes or its mouth (or any precise point on the robot) is likely less critical, as humans do not seem to excel at this themselves either, at least for inter-personal distances exceeding arm’s length. Humans seem biased towards perceiving eye contact, so what likely matters most is that the robot accurately estimates whether the human is attending to the robot at all. However, this presents a technical challenge. Estimating gaze direction from video is easier when the gaze is directed roughly towards the camera than for more eccentric gaze angles (e.g., Kellnhofer et al., 2019). Accurately estimating gaze direction in the world is thus more difficult than gaze toward the robot if the camera is mounted on the robot. Additional cameras in the world that serve as remote sensors for the social robot may be a potential solution to this problem.

A second implication of the human perceptual limits in gaze direction perception for social robotic interpretation concerns higher-level reasoning about human cognition, specifically theory of mind (see, e.g., Baron-Cohen, 1997; Frith & Frith, 2005, 2006). By understanding what humans can perceive from foveal versus peripheral vision, a robot can infer not only what a person is attending to themselves, but also what they are likely to be aware of regarding other humans in their immediate surroundings. This knowledge can guide predictions of future human behavior, for example, anticipating actions or communicative signals based on what the human knows or has seen, enabling more effective social interaction, particularly in multiparty interactions.

### Production

The implications of human perceptual limits for social robotic gaze production mirror those on the interpretation side. First, given human biases toward perceiving eye contact or being looked at under uncertainty, one may expect that if a robot already looks roughly in the right direction, this suffices for perceiving direct gaze. If humans cannot accurately distinguish being looked at in the eyes or mouth in a particular interaction context, there is no immediate need to look accurately at that area. However, it has been shown that in conversational interactions, gaze to the eyes or mouth is related to, for example, speaking or listening (Holleman et al., 2021), and gaze may thus be expected to alternate between these areas during interactions. Thus, timing or dynamic changes in gaze location can be optimized for, rather than the absolute accuracy of gaze location. For example, Pan et al. (2020) suggested that generating saccades may already produce the illusion of vergence and being looked at, rather than through. Note, however, that perceptual research has shown that humans may accept a considerable range of vergence angles as direct gaze (Linke & Horstmann, 2022).

A second implication is that robots may not need to produce overt gaze shifts to attend to or interpret objects or events that humans can also perceive from the periphery. For example, a human’s shift in body or head orientation 90$^{\circ }$ away from the robot’s gaze direction need not evoke a gaze shift, but can be readily used to update the robot’s model of the human’s locus of spatial attention (cf. Loomis et al., 2008). Thus, knowledge of human perceptual limits may inform which gaze functions to implement in social robots or which gaze behaviors to execute in situations of competing demands.

### Towards human-informed gaze perception for social robotics

The principles of human-informed gaze perception, interpretation, and production are visualized in Fig. 2. In this scenario, the robot (R) and human 2 (H$_2$) maintain direct gaze, human 1 (H$_1$) makes a gaze shift from the robot to a location in the background with their eyes only, and human 3 (H$_3$) makes a combined eye-head movement from the robot to H$_1$. As a first step, one can ask what the robot ought to perceive under the assumption that the robot perceives as well as a human would (technical feasibility notwithstanding), and without making gaze movements itself. Based on human perceptual limits, the robot can be expected to perceive the direct gaze of H$_2$, as it is in foveal vision. Based on studies of peripheral perception, H$_3$’s gaze shift should also be perceivable, and could be attributed as a gaze shift towards H$_1$. However, H$_1$’s gaze shift may not be perceivable. Thus, the robot can update its knowledge of H$_3$’s gaze direction with reasonable certainty, but may fail to recognize H$_1$’s new gaze location in the world. Through modeling of uncertainty about such gaze states (cf. Hayhoe & Ballard, 2014), H$_1$ may in time be chosen as the next gaze target of the robot.

Applying the principles of the theory of mind to the robot, one can also ask what the robot may believe the human, specifically H$_2$, to know about the current state of the interaction. Taking the same scenario and applying the principles of human perceptual limits, the robot may believe H$_2$ can perceive the robot’s direct gaze, as well as H$_3$’s gaze shift from the robot to H$_1$ from H$_2$’s periphery. Like the robot, H$_2$ cannot perceive H$_1$’s gaze shift. Note that the robot’s theory of H$_2$’s mind is limited by its own perception. If the robot is not able to perceive H$_1$’s gaze shift, neither can it attribute knowledge of that gaze shift to H$_2$’s knowledge state. Should H$_1$’s gaze shift have been big enough to be perceivable by the robot, the robot should still assume H$_2$ cannot perceive it, given the relative positioning and orientation of H$_1$ and H$_2$. Thus, a theory of mind process could be designed to be limited by the assumed perceptual capabilities of the human, even though the robot might be able to outperform the human, e.g., with additional sensors that the human does not have.

## Adaptive gaze for social robotics

Gaze behavior cannot be explained from perceptual necessity alone, as has been emphasized in the context of gaze to faces (Hessels, 2020). For example, just because head direction can be estimated from 90$^{\circ }$ in the periphery does not mean that people do not shift their gaze to that person if they have the flexibility to do so. Similarly, just because looking someone in the eyes is not perceptually necessary to the interaction at a given point in time, it does not mean humans do not look there. In essence, gaze direction and perception are related but not identical (see also the discussion of the eye-mind hypothesis in Hessels et al., 2025b). It is therefore not surprising that substantial individual differences in gaze behavior have been observed. For example, individual differences in gaze to facial features are large, both in studies with pictures of faces (Peterson & Eckstein, 2013; Mehoudar et al., 2014; Arizpe et al., 2017) and for real interactions (Peterson et al., 2016; Rogers et al., 2018), which may reflect object-generic biases (Broda & De Haas, 2024). Such differences have also been observed for whether people tend to look at others or where people look on bodies during brief passing encounters (Hessels et al., 2020). Individual differences in gaze to faces have been further related to clinical traits of autism or social anxiety (Wieser et al., 2009; Freeth et al., 2013; Jones & Klin, 2013; Hessels et al., 2018; Chen et al., 2022) with no unequivocal relation to social difficulty, or to cultural norms (Haensel et al., 2022; Hessels et al., 2025a).

The evidence reviewed above primarily concerns human gaze production, that is, where and when people choose to look. However, gaze processing is likewise shaped by social characteristics of both the observer and the observed individual. A substantial body of work demonstrates that gaze-triggered shifts of attention are modulated by social features of the interaction partner and the observer themselves (Dalmaso et al., 2020). For example, gaze-cueing effects vary as a function of the observer’s gender (Bayliss et al., 2005) and age (Kuhn et al., 2015), social attributes of the observed individual, such as dominance (Jones et al., 2010) and social status (Dalmaso et al., 2012), as well as the relation between observer and observed individual (Dalmaso et al., 2020). Thus, gaze perception reflects an interaction between perceptual signals and social context rather than a fixed stimulus–response mapping.

Together, these findings highlight the flexibility of gaze behavior beyond strict perceptual constraints or context-generic, automatic responses to gaze cues. Against that background, social robots may need to adapt both to individual differences in how gaze is interpreted and to produce gaze behavior that is representative of a broad range of social identities and roles. Ignoring these social modulators risks designing systems whose gaze is perceptually accurate yet socially inappropriate or ambiguous.

### Interpretation

Given the substantial individual, clinical, and cultural variability in gaze behavior that is expected in the context of human–robot interaction, social robots face the complex challenge of interpreting eye contact and other aspects of gaze behavior. A key challenge for robots is to distinguish perceptually critical looks, which are directly informative about a person’s ongoing behavior or intention, from noncritical ones that may reflect individual biases, cultural norms, or clinical traits such as autism or social anxiety. The former may be equally predictive across individuals, e.g., visually guided action, while the latter may reveal substantial variability. This distinction is essential not only for intention prediction and behavior understanding, but also for preparing adaptive responses: a robot may need to flexibly adjust its interpretation and reactions depending on the context, rather than assuming a one-size-fits-all rule for gaze, for example, about the meaning of eye contact avoidance. Such context-sensitive interpretation could be achieved either by integrating prior knowledge about the user’s cultural or clinical background or by learning online from ongoing interactions. However, we deem it unlikely that the meaning of a particular gaze may always be unequivocal.

### Production

In addition to interpreting the varied patterns of gaze behavior that may be observed across individuals in human–robot interaction, the large individual differences in gaze behavior are also fundamental to the production of gaze behavior for the robot. If robots produce gaze behavior that is based on empirical rules obtained from human studies (e.g., Huang & Mutlu, 2012), the robot naturally defaults to the empirical mean, or to the characteristics of the training data. While that may yield believable gaze behavior from a robot, it begs the question of what kind of agent is thereby portrayed. Recent studies have attempted to recreate different personalities through gaze behavior, by modeling after individuals with, e.g., different levels of extraversion (Andrist et al., 2015; Shintani et al., 2024).

As robots’ gaze becomes more individualized, modeled after humans with particular behavioral profiles, the question of how gaze behavior is determined in the interaction becomes more prominent. How do humans with different personalities, from different cultures, or of different clinical statuses interact with each other and adjust their gaze behavior to each other? Such adaptation is naturally going to be relevant for human–robot interaction as well. However, given that high-quality eye-tracking studies in human–human interaction have become prominent only in the last decade or two (e.g., Brône & Oben, 2018; Hessels, 2020; Valtakari et al., 2021), much of the empirical groundwork on this topic still has to be covered. In our view, interdisciplinary efforts on human–human interaction and social robotics may play a crucial role in advancing the research field.

## Closing the loop: From human to robot to theory

The central question of this perspective is how social robots can be designed to use gaze in ways that support believable and appropriate human–robot interaction. We have argued that researchers should move beyond the traditional “eye-mind” assumption, and have identified three core challenges to consider from the perspective of human–human interaction for the interpretation and production of gaze patterns in social robotics.

First, social robotics can serve as a mirror for psychological theory by explicitly addressing the dual functions of gaze as both a perceptual system and a potential communicative signal, which so far has been poorly understood in human–human interactions. Crucially, social robots may allow experimental manipulation of perceptual and communicative roles that are not feasible in human interactions. This opens the door to more nuanced theories of gaze that move beyond simple dichotomies like overt/covert attention or perceptual/communicative functions of gaze. Rather, it pushes us forward towards gaze models that capture dynamic, concurrent, and sometimes conflicting functions.

Second, understanding how humans perceive gaze direction is essential for building social robots that can both interpret and produce meaningful gaze behavior. Although human perception studies have shown remarkable sensitivity to fine-grained gaze cues, they also reveal biases, limits, and the fact that gaze shifts are not always perceptually necessary, even when they may be commonly observed. For social robotics, these findings highlight open questions about when robots should signal gaze precisely or how they ought to interpret that of humans, as well as how higher-level reasoning such as theory of mind can be implemented. Such theory-of-mind models may prove useful for characterizing and predicting gaze behavior in human–human interactions.

Third, we have highlighted that human gaze behavior is shaped not only by perceptual constraints but also by large individual, cultural, and clinical differences. As such, social robots may be expected to understand such wide variability, as well as demonstrate adaptive gaze behavior rather than relying on fixed, one-size-fits-all behaviors. Social robotics offers a unique testbed for advancing our understanding of gaze in human–human interactions, because robots allow systematic manipulation of gaze behaviors in controlled yet interactive settings. This makes it possible to begin disentangling individual from dyadic behaviors in a way that is difficult in human–human studies, where interactional measures reflect contributions from both partners. Such approaches are particularly valuable for studying clinical conditions like autism or social anxiety, where individual traits, family context, and so forth, can otherwise be hard to separate.

The conceptual advance we propose lies in treating social robotics as an experimental lens for re-examining long-standing psychological theories of gaze through a perceptual perspective. Specifically, we argue that the dual-function view of gaze (perceptual versus communicative), the empirical constraints and biases governing gaze direction perception, and the broad individual and cultural variability in gaze use can all be unified within a single perspective that views social gaze as a soft-constraints gaze inference (and gaze production) process under uncertainty. Perceptually speaking, gaze behavior may reflect the continuous inference about where others are looking and why, which is constrained by both biological and contextual factors. Communicatively speaking, gaze behavior is flexible enough to express intentions, emotions, and represent individual communication styles and biases. Gaze is thus not a fixed signal but a probabilistic behavior shaped by potentially competing goals and tolerances for ambiguity (cf. Hayhoe & Ballard, 2005, 2014). Thinking of gaze as inference under soft constraints (see e.g., Gray et al., 2006) explains how the same perceptual system can support both visually driven exploration and socially meaningful communication, and why substantial inter-individual and cultural variability is observed. This perspective extends classic foundations in social and perceptual psychology, explicitly incorporating them into computational and robotic architectures that make perceptual assumptions testable. Robotic implementations, in turn, enable systematic manipulation of the constraints and uncertainties that shape gaze, revealing how perceptual mechanisms may give rise to social meaning.

More specifically, robotic systems make perceptual and communicative hypotheses about gaze empirically tractable in ways that are rarely possible in studies of human–human interaction. By explicitly manipulating perceptual constraints, such as the coupling between gaze direction and sensor input, or the uncertainty in a robot’s gaze estimation, researchers can test how perceptual structure shapes communicative interpretation. Similarly, robots can selectively exaggerate, omit, or decouple gaze cues from their behavior to isolate perceptual aspects from communicative aspects of gaze. This may allow empirical tests of boundary conditions of classic model components such as the eye-direction detector and shared-attention mechanism. These experimental manipulations allow the formulation of genuinely new tests of psychological theories: for instance, can inferred agency emerge from perceptual contingency alone, and if so, when, and how do perceptual biases constrain social meaning attribution? In this way, social robotics functions not merely as a demonstration platform but as an experimental methodology for theory building in perception and social cognition. We hope that this perspective and its empirical implementations serve as a bridge between perceptual science and social robotics, motivating principled models that explain not only *where* agents look, but *why* gaze acquires meaning in interaction.

While black-box machine learning approaches have shown promise in both assessing human behavior and generating intuitive and lifelike behaviors, they often lack insight into the underlying mechanisms of perception, reasoning, or adaptation. These models can recognize or mimic human behaviors, but without a clear grounding or interpretive framework, they do not explain why a behavior occurs. This makes their outputs difficult to interpret, personalize, or align with human expectations, particularly in social robotics, where agents differ from humans not only in appearance and motor degrees of freedom, but also in how they perceive the world and express themselves. In this context, systems must not only recognize a human user’s state, but also generate behaviors that are understandable and socially meaningful. In the short term, black-box methods may suffice to simulate interaction. In the long run, however, they risk becoming bottlenecks as we pursue more flexible, trustworthy, and socially aligned systems. To move beyond opaque, surface-level replication of human behavior, we must invest in principled models, grounded in perception, cognition, and individual differences in human behavior. These models not only help clarify how behavior is generated, but also provide the foundation for systems that can generalize, individually adapt, and socially align within complex human social environments.

In our view, perceptual and cognitive research provide the necessary foundation for principled models of gaze behavior in social robotics. In contrast, the engineering challenges in social robotics provide the ideal testbed for implementing, validating, and refining such models to ensure they have practical utility. As social scientists, perception scientists, computer scientists, and engineers develop, build, and validate machines that must know when and where to look, we may better understand how and why humans do the same.

## Data Availability

Not applicable.

## Declarations

### Open Practices Statement

There are no data or code associated with the present article.

## References

[CR1] Admoni, H., & Scassellati, B. (2017). Social eye gaze in human-robot interaction: A review. *Journal of Human-Robot Interaction,**6*(1), 25–63.

[CR2] Andrist, S., Mutlu, B., & Tapus, A. (2015). Look like me: Matching Robot Personality via Gaze to Increase Motivation. In *Proceedings of the 33rd Annual ACM Conference on Human Factors in Computing Systems,* pages 3603–3612, Seoul, Republic of Korea. ACM.

[CR3] Anstis, S. M., Mayhew, J. W., & Morley, T. (1969). The Perception of Where a Face or Television ‘Portrait’ Is Looking. *The American Journal of Psychology,**82*(4), 474–489. 5398220

[CR4] Argyle, M., & Dean, J. (1965). Eye-contact, distance and affiliation. *Sociometry,**28*(3), 289–304. 14341239

[CR5] Argyle, M., & Cook, M. (1976). *Gaze and Mutual Gaze*. Cambridge, England: Cambridge University Press.

[CR6] Arizpe, J., Walsh, V., Yovel, G., & Baker, C. I. (2017). The categories, frequencies, and stability of idiosyncratic eye-movement patterns to faces. *Vision Research,**141*, 191–203. 27940212 10.1016/j.visres.2016.10.013 PMC5474224

[CR7] Baron-Cohen, S. (1997). Mindblindness: An Essay on Autism and Theory of Mind. MIT Press.

[CR8] Bayliss, A. P., Di Pellegrino, G., & Tipper, S. P. (2005). Sex differences in eye gaze and symbolic cueing of attention. *The Quarterly Journal of Experimental Psychology Section A,**58*(4), 631–650. 10.1080/02724980443000124 16104099

[CR9] Belardinelli, A. (2024). Gaze-based intention estimation: Principles, methodologies, and applications in HRI. *ACM Transactions on Human-Robot Interaction,**13*(3), 1–30.

[CR10] Botvinick, M., & Plaut, D. C. (2004). Doing without schema hierarchies: A recurrent connectionist approach to normal and impaired routine sequential action. *Psychological Review,**111*(2), 395–429. 15065915 10.1037/0033-295X.111.2.395

[CR11] Boucher, J.-D., Pattacini, U., Lelong, A., Bailly, G., Elisei, F., Fagel, S., . . . Ventre-Dominey, J. (2012). I reach faster when i see you look: Gaze effects in human–human and human-robot face-to-face cooperation. *Frontiers in Neurorobotics*, 6. 10.3389/fnbot.2012.00003 PMC3342577 22563315

[CR12] Bringmann, A., Syrbe, S., Görner, K., Kacza, J., Francke, M., Wiedemann, P., & Reichenbach, A. (2018). The primate fovea: Structure, function and development. *Progress in Retinal and Eye Research,**66*, 49–84. 29609042 10.1016/j.preteyeres.2018.03.006

[CR13] Broda, M. D., & De Haas, B. (2024). Individual differences in human gaze behavior generalize from faces to objects. In *Proceedings of the National Academy of Sciences,* *121*(12), Article e2322149121. 10.1073/pnas.2322149121 PMC10963009 38470925

[CR14] Brône, G. & Oben, B., (Eds.) (2018). *Eye-Tracking in Interaction*. Studies on the Role of Eye Gaze in Dialogue. John Benjamins Publishing Company, Amsterdam / Philadelphia.

[CR15] Chen, M. (2002). Leveraging the Asymmetric Sensitivity of Eye Contact for Videoconference. *CHI ’02 Proceedings of the SIGCHI Conference on Human Factors in Computing Systems,* *4*(1), 49–56.

[CR16] Chen, J., van den Bos, E., Karch, J. D., & Westenberg, P. M. (2022). Social anxiety is related to reduced face gaze during a naturalistic social interaction. *Anxiety, Stress, & Coping,* pages 1–15. 10.1080/10615806.2022.2125961 36153759

[CR17] Cihodaru-Ștefanache, Ș, & Podina, I. R. (2025). The uncanny valley effect in embodied conversational agents: A critical systematic review of attractiveness, anthropomorphism, and uncanniness. *Frontiers in Psychology,* *16*, 1625984. 10.3389/fpsyg.2025.1625984 PMC12493983 41050809

[CR18] Clark, H. H. (1996). *Using Language*. Cambridge University Press.

[CR19] Cline, M. G. (1967). The perception of where a person is looking. *The American Journal of Psychology,**80*(1), 41–50. 6036357

[CR20] Cooper, R., & Shallice, T. (2000). Contention scheduling and the control of routine activities. *Cognitive Neuropsychology,**17*(4), 297–338. 20945185 10.1080/026432900380427

[CR21] Dalmaso, M., Pavan, G., Castelli, L., & Galfano, G. (2012). Social status gates social attention in humans. *Biology Letters,**8*(3), 450–452. 22090207 10.1098/rsbl.2011.0881 PMC3367721

[CR22] Dalmaso, M., Castelli, L., & Galfano, G. (2020). Social modulators of gaze-mediated orienting of attention: A review. *Psychonomic Bulletin & Review,**27*(5), 833–855. 32291650 10.3758/s13423-020-01730-x

[CR23] Driver, J., Davis, G., Ricciardelli, P., Kidd, P., Maxwell, E., & Baron-Cohen, S. (1999). Gaze perception triggers reflexive visuospatial orienting. *Visual Cognition,**6*(5), 509–540.

[CR24] Emery, N. J. (2000). The eyes have it: The neuroethology, function and evolution of social gaze. *Neuroscience & Biobehavioral Reviews,**24*, 581–604. 10940436 10.1016/s0149-7634(00)00025-7

[CR25] Fang, Y., Pérez-Molerón, J. M., Merino, L., Yeh, S.-L., Nishina, S., & Gomez, R. (2024). Enhancing social robot’s direct gaze expression through vestibulo-ocular movements. *Advanced Robotics,**38*(19–20), 1457–1469.

[CR26] Flanagan, J. R., & Johansson, R. S. (2003). Action plans used in action observation. *Nature,**424*(6950), 769–771. 12917683 10.1038/nature01861

[CR27] Florey, J., Clifford, C. W. G., Dakin, S. C., & Mareschal, I. (2015). Peripheral processing of gaze. *Journal of Experimental Psychology: Human Perception and Performance,**41*(4), 1084–1094. 26010593 10.1037/xhp0000068

[CR28] Foulsham, T., & Kingstone, A. (2025). Covert orienting: The dark matter of social attention. *Trends in Cognitive Sciences*. 10.1016/j.tics.2025.05.001 40410094

[CR29] Freeth, M., Foulsham, T., & Kingstone, A. (2013). What affects social attention? Social presence, eye contact and autistic traits. *PLOS One,* *8*(1), Article e53286. 10.1371/journal.pone.0053286 PMC3541232 23326407

[CR30] Frischen, A., Bayliss, A. P., & Tipper, S. P. (2007). Gaze cueing of attention: Visual attention, social cognition, and individual differences. *Psychological Bulletin,**133*(4), 694–724. 17592962 10.1037/0033-2909.133.4.694 PMC1950440

[CR31] Frith, C., & Frith, U. (2005). Theory of mind. *Current Biology,**15*(17), R644–R645. 16139190 10.1016/j.cub.2005.08.041

[CR32] Frith, C. D., & Frith, U. (2006). The Neural basis of mentalizing. *Neuron,**50*(4), 531–534. 16701204 10.1016/j.neuron.2006.05.001

[CR33] Gallup, A. C., Chong, A., & Couzin, I. D. (2012). The directional flow of visual information transfer between pedestrians. *Biology Letters,**8*(4), 520–522. 22456331 10.1098/rsbl.2012.0160 PMC3391476

[CR34] Gamer, M., & Hecht, H. (2007). Are you looking at me? Measuring the cone of gaze. *Journal of Experimental Psychology: Human Perception and Performance,**33*(3), 705–715. 17563231 10.1037/0096-1523.33.3.705

[CR35] García-Martínez, J., Gamboa-Montero, J. J., Castillo, J. C., Castro-González, Á., & Salichs, M. A. (2025). Implementation of a biologically inspired responsive joint attention system for a social robot. *Advanced Intelligent Systems,**7*(6), 2400650.

[CR36] Gibson, J. J., & Pick, A. D. (1963). Perception of another person’s looking behavior. *The American Journal of Psychology,**76*(3), 386–394. 13947729

[CR37] Gobel, M. S., Kim, H. S., & Richardson, D. C. (2015). The dual function of social gaze. *Cognition,**136*, 359–364. 25540833 10.1016/j.cognition.2014.11.040

[CR38] Gray, W. D., Sims, C. R., Fu, W.-T., & Schoelles, M. J. (2006). The soft constraints hypothesis: A rational analysis approach to resource allocation for interactive behavior. *Psychological Review,**113*(3), 461–482. 16802878 10.1037/0033-295X.113.3.461

[CR39] Haefflinger, L., Elisei, F., Gerber, S., Bouchot, B., Vigne, J.-P., & Bailly, G. (2023). On the benefit of independent control of head and eye movements of a social robot for multiparty human-robot interaction. In M. Kurosu & A. Hashizume (Eds.), *Human-Computer Interaction* (Vol. 14011, pp. 450–466). Cham: Springer Nature Switzerland.

[CR40] Haensel, J. X., Smith, T. J., & Senju, A. (2022). Cultural differences in mutual gaze during face-to-face interactions: A dual head-mounted eye-tracking study. *Visual Cognition,**30*(1–2), 100–115.

[CR41] Hanifi, S., Maiettini, E., Lombardi, M., & Natale, L. (2023). iCub Detecting Gazed Objects: A Pipeline Estimating Human Attention. 10.3389/frobt.2024.1346714 PMC11524796 39483489

[CR42] Hanna, J. E., & Brennan, S. E. (2007). Speakers’ eye gaze disambiguates referring expressions early during face-to-face conversation. *Journal of Memory and Language,**57*(4), 596–615.

[CR43] Hayhoe, M. (2000). Vision using routines: A functional account of vision. *Visual Cognition,**7*(1–3), 43–64.

[CR44] Hayhoe, M. M. (2017). Vision and action. *Annual Review of Vision Science,**3*, 389–413. 28715958 10.1146/annurev-vision-102016-061437

[CR45] Hayhoe, M., & Ballard, D. (2005). Eye movements in natural behavior. *Trends in Cognitive Sciences,**9*(4), 188–194. 15808501 10.1016/j.tics.2005.02.009

[CR46] Hayhoe, M., & Ballard, D. (2014). Modeling task control of eye movements. *Current Biology,**24*(13), R622–R628. 25004371 10.1016/j.cub.2014.05.020 PMC4150691

[CR47] Heron, J. (1970). The phenomenology of social encounter: The gaze. *Philosophy and Phenomenological Research,**31*(2), 243–264.

[CR48] Hessels, R. S. (2020). How does gaze to faces support face-to-face interaction? A review and perspective. *Psychonomic Bulletin & Review,**27*, 856–881. 32367351 10.3758/s13423-020-01715-w PMC7547045

[CR49] Hessels, R. S., Holleman, G. A., Cornelissen, T. H. W., Hooge, I. T. C., & Kemner, C. (2018). Eye contact takes two - autistic and social anxiety traits predict gaze behavior in dyadic interaction. *Journal of Experimental Psychopathology*, (April-June):1–17.

[CR50] Hessels, R. S., Holleman, G. A., Kingstone, A., Hooge, I. T. C., & Kemner, C. (2019). Gaze allocation in face-to-face communication is affected primarily by task structure and social context, not stimulus-driven factors. *Cognition,**184*, 28–43. 30557748 10.1016/j.cognition.2018.12.005

[CR51] Hessels, R. S., Benjamins, J. S., van Doorn, A. J., Koenderink, J. J., Holleman, G. A., & Hooge, I. T. C. (2020). Looking behavior and potential human interactions during locomotion. *Journal of Vision,**20*(10), 5. 33007079 10.1167/jov.20.10.5 PMC7545070

[CR52] Hessels, R. S., Iwabuchi, T., Niehorster, D. C., Funawatari, R., Benjamins, J. S., Kawakami, S., . . . Senju, A. (2025a). Gaze behavior in face-to-face interaction: A cross-cultural investigation between Japan and the Netherlands. *Cognition,**263*, Article 106174. 10.1016/j.cognition.2025.106174 40424776

[CR53] Hessels, R. S., Nuthmann, A., Nyström, M., Andersson, R., Niehorster, D. C., & Hooge, I. T. C. (2025b). The fundamentals of eye tracking part 1: The link between theory and research question. *Behavior Research Methods,* *57*(1), 16. 10.3758/s13428-024-02544-8 PMC11638287 39668288

[CR54] Ho, S., Foulsham, T., & Kingstone, A. (2015). Speaking and listening with the eyes: Gaze signaling during dyadic interactions. *PLOS One,* *10*(8), Article e0136905. 10.1371/journal.pone.0136905 PMC4550266 26309216

[CR55] Holleman, G. A., Hooge, I. T. C., Huijding, J., Deković, M., Kemner, C., & Hessels, R. S. (2021). Gaze and speech behavior in parent–child interactions: The role of conflict and cooperation. *Current Psychology*.

[CR56] Honma, M., Tanaka, Y., Osada, Y., & Kuriyama, K. (2012). Perceptual and not physical eye contact elicits pupillary dilation. *Biological Psychology,**89*(1), 112–116. 21982748 10.1016/j.biopsycho.2011.09.015

[CR57] Horstmann, G., & Linke, L. (2021). Examining gaze cone shape and size. *Perception,**50*(12), 1056–1065. 34841983 10.1177/03010066211059930

[CR58] Horstmann, G., & Linke, L. (2025). The effect of distance on the overestimation of gaze direction. *Journal of Experimental Psychology: Human Perception and Performance,**51*(2), 260–281. 39913495 10.1037/xhp0001295

[CR59] Huang, C.-M., & Mutlu, B. (2012). Robot behavior toolkit: Generating effective social behaviors for robots. In *Proceedings of the 7th Annual ACM/IEEE International Conference on Human-Robot Interaction - HRI ’12,* pages 25–32, Boston, Massachusetts, USA. ACM Press.

[CR60] Jones, B. C., DeBruine, L. M., Main, J. C., Little, A. C., Welling, L. L. M., Feinberg, D. R., & Tiddeman, B. P. (2010). Facial cues of dominance modulate the short-term gaze-cuing effect in human observers. *Proceedings of the Royal Society B: Biological Sciences,**277*(1681), 617–624. 10.1098/rspb.2009.1575 PMC2842686 19864283

[CR61] Jones, W., & Klin, A. (2013). Attention to eyes is present but in decline in 2-6-month-old infants later diagnosed with autism. *Nature,**504*, 427–431. 24196715 10.1038/nature12715 PMC4035120

[CR62] Jongerius, C., Hessels, R. S., Romijn, J. A., Smets, E. M. A., & Hillen, M. A. (2020). The measurement of eye contact in human interactions: A scoping review. *Journal of Nonverbal Behavior*.

[CR63] Kellnhofer, P., Recasens, A., Stent, S., Matusik, W., & Torralba, A. (2019). Gaze360: Physically unconstrained gaze estimation in the wild. In *Proceedings of the IEEE/CVF International Conference on Computer Vision (ICCV)*.

[CR64] Kendon, A. (1967). Some functions of gaze-direction in social interaction. *Acta Psychologica,**26*, 22–63. 6043092 10.1016/0001-6918(67)90005-4

[CR65] Kingstone, A. (2009). Taking a real look at social attention. *Current Opinion in Neurobiology,**19*, 52–56. 19481441 10.1016/j.conb.2009.05.004

[CR66] Kleinke, C. L. (1986). Gaze and eye contact: A research review. *Psychological Bulletin,**100*(1), 78–100. 3526377

[CR67] Kluttz, N. L., Mayes, B. R., West, R. W., & Kerby, D. S. (2009). The effect of head turn on the perception of gaze. *Vision Research,**49*(15), 1979–1993. 19467254 10.1016/j.visres.2009.05.013

[CR68] Krüger, M., Oshima, Y., & Fang, Y. (2026). Virtual reflections on a dynamic 2-D eye model improve spatial reference identification. *IEEE Transactions on Human-Machine Systems*, pages 1–10.

[CR69] Kuhn, G., Pagano, A., Maani, S., & Bunce, D. (2015). Age-related decline in the reflexive component of overt gaze following. *Quarterly Journal of Experimental Psychology,**68*(6), 1073–1081. 10.1080/17470218.2014.975257 25397861

[CR70] Land, M., Mennie, N., & Rusted, J. (1999). The roles of vision and eye movements in the control of activities of daily living. *Perception,**28*(11), 1311–1328. 10755142 10.1068/p2935

[CR71] Langton, S. R. H., Honeyman, H., & Tessler, E. (2004). The influence of head contour and nose angle on the perception of eye-gaze direction. *Perception & Psychophysics,**66*(5), 752–771. 15495901 10.3758/bf03194970

[CR72] Lavit Nicora, M., Prajod, P., Mondellini, M., Tauro, G., Vertechy, R., André, E., & Malosio, M. (2024). Gaze detection as a social cue to initiate natural human-robot collaboration in an assembly task. *Frontiers in Robotics and AI,**11*, 1394379. 39086514 10.3389/frobt.2024.1394379 PMC11288793

[CR73] Linke, L., & Horstmann, G. (2022). How vergence influences the perception of being looked at. *Perception,**51*(11), 789–803. 36062732 10.1177/03010066221122359

[CR74] Loomis, J. M., Kelly, J. W., Pusch, M., Bailenson, J. N., & Beall, A. C. (2008). Psychophysics of perceiving eye-gaze and head direction with peripheral vision: Implications for the dynamics of eye-gaze behavior. *Perception,**37*(9), 1443–1457. 18986070 10.1068/p5896

[CR75] Loschky, L. C., Szaffarczyk, S., Beugnet, C., Young, M. E., & Boucart, M. (2019). The contributions of central and peripheral vision to scene-gist recognition with a 180 visual field. *Journal of Vision,**19*(5), 1–21. 10.1167/19.5.15 31100131

[CR76] Macdonald, R. G., & Tatler, B. W. (2013). Do as eye say: Gaze cueing and language in a real-world social interaction. *Journal of Vision,**13*(4), 1–12. 10.1167/13.4.6 23479476

[CR77] Maran, T., Furtner, M., Liegl, S., Ravet-Brown, T., Haraped, L., & Sachse, P. (2021). Visual attention in real-world conversation: gaze patterns are modulated by communication and group size. *Applied Psychology,**70*(4), 1602–1627.

[CR78] Mareschal, I., Calder, A. J., & Clifford, C. W. G. (2013). Humans have an expectation that gaze is directed toward them. *Current Biology,**23*(8), 717–721. 23562265 10.1016/j.cub.2013.03.030 PMC3918857

[CR79] Mareschal, I., Calder, A. J., Dadds, M. R., & Clifford, C. W. G. (2013). Gaze categorization under uncertainty: Psychophysics and modeling. *Journal of Vision,**13*(5), 18–18. 23608340 10.1167/13.5.18

[CR80] Mehoudar, E., Arizpe, J., Baker, C. I., & Yovel, G. (2014). Faces in the eye of the beholder: Unique and stable eye scanning patterns of individual observers. *Journal of Vision,**14*(7), 6. 25057839 10.1167/14.7.6 PMC4062043

[CR81] Millen, A. E., & Hancock, P. J. B. (2019). Eye see through you! Eye tracking unmasks concealed face recognition despite countermeasures. *Cognitive Research: Principles and Implications,**4*(1), 23. 31388791 10.1186/s41235-019-0169-0 PMC6684707

[CR82] Moon, Aj., Troniak, D. M., Gleeson, B., Pan, M. K., Zheng, M., Blumer, B. A., . . . Croft, E. A. (2014). Meet me where I’m gazing: How shared attention gaze affects human-robot handover timing. In *Proceedings of the 2014 ACM/IEEE International Conference on Human–Robot Interaction,* pages 334–341, Bielefeld Germany. ACM.

[CR83] Mutlu, B., Shiwa, T., Kanda, T., Ishiguro, H., & Hagita, N. (2009). Footing in human-robot conversations: How robots might shape participant roles using gaze cues. In *HRI ’09*, pages 61–68, New York, New York, USA. SIGCHI, ACM Special Interest Group on Computer–Human Interaction.

[CR84] Mwangi, E., Barakova, E. I., Díaz-Boladeras, M., Mallofré, A. C., & Rauterberg, M. (2018). Directing attention through gaze hints improves task solving in human-humanoid interaction. *International Journal of Social Robotics,**10*(3), 343–355. 10.1007/s12369-018-0473-8 PMC6438392 30996753

[CR85] Nasiopoulos, E., Risko, E. F., & Kingstone, A. (2015). Social Attention, Social Presence, and the Dual Function of Gaze. In A. Puce & B. I. Bertenthal (Eds.), *The Many Faces of Social Attention* (pp. 129–155). Cham: Springer International Publishing.

[CR86] Pan, M. K., Choi, S., Kennedy, J., McIntosh, K., Zamora, D. C., Niemeyer, G., . . . Christensen, D. (2020). Realistic and Interactive Robot Gaze. In *2020 IEEE/RSJ International Conference on Intelligent Robots and Systems (IROS),* pages 11072–11078, Las Vegas, NV, USA. IEEE.

[CR87] Pelz, J. B., & Canosa, R. (2001). Oculomotor behavior and perceptual strategies in complex tasks. *Vision Research,**41*, 3587–3596. 11718797 10.1016/s0042-6989(01)00245-0

[CR88] Peterson, M. F., & Eckstein, M. P. (2013). Individual differences in eye movements during face identification reflect observer-specific optimal points of fixation. *Psychological Science,**24*(7), 1216–1225. 23740552 10.1177/0956797612471684 PMC6590077

[CR89] Peterson, M. F., Lin, J., Zaun, I., & Kanwisher, N. (2016). Individual differences in face-looking behavior generalize from the lab to the world. *Journal of Vision,**16*(7), 12. 27191940 10.1167/16.7.12

[CR90] Risko, E. F., Richardson, D. C., & Kingstone, A. (2016). Breaking the fourth wall of cognitive science: Real-world social attention and the dual function of gaze. *Current Directions in Psychological Science,**25*(1), 70–74.

[CR91] Robinson, N., Tidd, B., Campbell, D., Kulić, D., & Corke, P. (2023). Robotic vision for human-robot interaction and collaboration: A survey and systematic review. *ACM Transactions on Human-Robot Interaction,**12*(1), 1–66.

[CR92] Rogers, S. L., Speelman, C. P., Guidetti, O., & Longmuir, M. (2018). Using dual eye tracking to uncover personal gaze patterns during social interaction. *Scientific Reports,**8*, 4271. 29523822 10.1038/s41598-018-22726-7 PMC5844880

[CR93] Rosenholtz, R. (2016). Capabilities and limitations of peripheral vision. *Annual Review of Vision Science,**2*(1), 437–457. 28532349 10.1146/annurev-vision-082114-035733

[CR94] Ruesch, J., Lopes, M., Bernardino, A., Hornstein, J., Santos-Victor, J., & Pfeifer, R. (2008). Multimodal saliency-based bottom-up attention: A framework for the humanoid robot iCub. In *2008 IEEE International Conference on Robotics and Automation,* pages 962–967, Pasadena, CA, USA. IEEE.

[CR95] Ruhland, K., Peters, C. E., Andrist, S., Badler, J. B., Badler, N. I., Gleicher, M., . . . McDonnell, R. (2015). A review of eye gaze in virtual agents, social robotics and HCI: Behaviour generation, user interaction and perception. *Computer Graphics Forum,* *34*(6), 299–326.

[CR96] Shintani, T., Ishi, C. T., & Ishiguro, H. (2024). Gaze modeling in multi-party dialogues and extraversion expression through gaze aversion control. *Advanced Robotics,**38*(19–20), 1470–1485.

[CR97] Sullivan, B., Ludwig, C. J. H., Damen, D., Mayol-Cuevas, W., & Gilchrist, I. D. (2021). Look-ahead fixations during visuomotor behavior: Evidence from assembling a camping tent. *Journal of Vision,**21*(3), 13. 33688920 10.1167/jov.21.3.13 PMC7961111

[CR98] Symons, L. A., Lee, K., Cedrone, C. C., & Nishimura, M. (2004). What are you looking at? Acuity for triadic eye gaze. *The Journal of General Psychology,**131*(4), 451–469. 15523825 PMC2564292

[CR99] Teufel, C., Alexis, D. M., Todd, H., Lawrance-Owen, A. J., Clayton, N. S., & Davis, G. (2009). Social cognition modulates the sensory coding of observed gaze direction. *Current Biology,**19*(15), 1274–1277. 10.1016/j.cub.2009.05.069 19559619

[CR100] Trick, S., Koert, D., Peters, J., & Rothkopf, C. A. (2019). Multimodal Uncertainty Reduction for Intention Recognition in Human-Robot Interaction. In *2019 IEEE/RSJ International Conference on Intelligent Robots and Systems (IROS),* pages 7009–7016, Macau, China. IEEE.

[CR101] Valtakari, N. V., Hooge, I. T. C., Viktorsson, C., Nyström, P., Falck-Ytter, T., & Hessels, R. S. (2021). Eye tracking in human interaction: Possibilities and limitations. *Behavior Research Methods,**53*, 1592–1608. 33409984 10.3758/s13428-020-01517-x PMC7787418

[CR102] Vater, C., Wolfe, B., & Rosenholtz, R. (2022). Peripheral vision in real-world tasks: A systematic review. *Psychonomic Bulletin & Review,**29*(5), 1531–1557. 35581490 10.3758/s13423-022-02117-w PMC9568462

[CR103] von Cranach, M., & Ellgring, J. H. (1973). Problems in the Recognition of Gaze Direction. *Social communication and movement: Studies of interaction and expression in man and chimpanzee,**4*, 419.

[CR104] Wieser, M. J., Pauli, P., Alpers, G. W., & Mühlberger, A. (2009). Is eye-to-eye contact really threatening and avoided in social anxiety?-An eye-tracking and psychophysiology study. *Journal of Anxiety Disorders,**23*, 93–103. 18534814 10.1016/j.janxdis.2008.04.004

[CR105] Wiese, E., Wykowska, A., Zwickel, J., & Müller, H. J. (2012). I see what you mean: How attentional selection is shaped by ascribing intentions to others. *PLoS ONE,* *7*(9), Article e45391. 10.1371/journal.pone.0045391 PMC3458834 23049794

[CR106] Wykowska, A., Chaminade, T., & Cheng, G. (2016). Embodied artificial agents for understanding human social cognition. *Philosophical Transactions of the Royal Society B: Biological Sciences,**371*(1693), 20150375. 10.1098/rstb.2015.0375 PMC4843613 27069052

[CR107] Xu, T. L., Zhang, H., & Yu, C. (2016). See you see me: The role of eye contact in multimodal human-robot interaction. *ACM Transactions on Interactive Intelligent Systems,**6*(1), 1–22. 10.1145/2882970 PMC5618804 28966875

[CR108] Zhang, R., Saran, A., Liu, B., Zhu, Y., Guo, S., Niekum, S., . . . Hayhoe, M. (2020). Human Gaze Assisted Artificial Intelligence: A Review. In *Proceedings of the 29th International Joint Conference on Artificial Intelligence,* pages 4951–4958, Yokohama, Japan. International Joint Conferences on Artificial Intelligence Organization. 10.24963/ijcai.2020/689 PMC7476326 32901189

[CR109] Zheleva, A., Hardeman, J., Durnez, W., Vanroelen, C., De Bruyne, J., Tutu, D. O., . . . Bombeke, K. (2023). The impact of eye gaze on social interactions of females in virtual reality: The mediating role of the uncanniness of avatars and the moderating role of task type. *Heliyon,* *9*(10), Article e20165. 10.1016/j.heliyon.2023.e20165 PMC10585207 37867871

